# Children's processing of emotion in ironic language

**DOI:** 10.3389/fpsyg.2013.00691

**Published:** 2013-10-08

**Authors:** Andrew Nicholson, Juanita M. Whalen, Penny M. Pexman

**Affiliations:** Language Processing Laboratory, Department of Psychology, University of CalgaryCalgary, AB, Canada

**Keywords:** verbal irony, eye gaze, empathy, children, speaker intent

## Abstract

In the present study we addressed two novel questions: (1) is children's irony appreciation and processing related to their empathy skills? and (2) is children's processing of a speaker's ironic meaning best explained by a modular or interactive theory? Participants were thirty-one 8- and 9-year-olds children. We used a variant of the visual world paradigm to assess children's processing of ironic and literal evaluative remarks; in this paradigm children's cognition is revealed through their actions and eye gaze. Results in this paradigm showed that children's irony appreciation and processing were correlated with their empathy development, suggesting that empathy or emotional perspective taking may be important for development of irony comprehension. Further, children's processing of irony was consistent with an interactive framework, in which children consider ironic meanings in the earliest moments, as speech unfolds. These results provide important new insights about development of this complex aspect of emotion recognition.

## Introduction

One of the challenges children face in developing emotion recognition is created by the fact that people often convey emotions indirectly, for example, through use of verbal irony. Verbal irony is a complex language device used by speakers who intend to convey an attitude that is of opposite valence to the meaning of the words spoken. Verbal irony is used for a wide variety of social purposes: to mute the effect of criticism in some instances and exacerbate the effect of criticism in others, to be humorous, and to manage relationships (see Pexman, 2008, for a review). Comprehension of irony requires the listener to use cues like tone of voice in order to infer the attitude and emotions of the speaker. This type of emotional perspective taking can be challenging for children and research suggests that irony appreciation develops over a long developmental window in middle childhood (Pexman and Glenwright, 2007). While there is as yet no developmental theory of irony appreciation, there is growing interest in the issue of how children reason about these statements.

As the most common type of verbal irony (Capelli et al., 1990), sarcasm involves a positively worded statement which is meant to be taken negatively. This language device is also referred to as an ironic criticism; for example, if you were to say “you're really good at that” when a friend fails at a sport. This form of irony is the focus of the current study.

Understanding irony is a complex process; it involves understanding the attitude and emotion of the speaker and also the impact they intend their statement to have. As such, irony is thought to be more difficult to process than other falsehoods, such as deception (Demorest et al., 1984). In order to appreciate verbal irony, one needs to understand that the speaker of the statement did not intend for the meaning of the words to be taken literally, that their belief about the situation was different than the literal meaning of their words, and that they deliberately intended for this falsehood to be detected. Finally, there needs to be an understanding of why the speaker would make such a statement. Children begin to appreciate these aspects of ironic intent between 6 and 10 years of age (Pexman and Glenwright, 2007; Filippova and Astington, 2008), with appreciation becoming more sophisticated through middle childhood and into adolescence (Demorest et al., 1984; Pexman et al., 2005). Previous research has mapped out the age range during which children's irony appreciation skills develop, but we know very little about the relationship of irony appreciation to other aspects of emotional development.

### The role of empathy

One goal of the present study was to assess the relationship of irony appreciation to empathy or emotional perspective taking skills. Empathy is the identification or reaction of one person to the experience of another and is thought to include a number of components, including empathic concern and perspective taking (e.g., Davis, 1983). Several researchers have proposed that emotional perspective taking, and in particular the ability to understand the emotions and attitudes behind others' mental states, is important to irony comprehension (Martin and McDonald, 2005; Shamay-Tsoory et al., 2005; Dennis et al., 2013). For instance, Shamay-Tsoory et al. examined irony appreciation in patients with brain damage to the right ventromedial prefrontal cortex (vmPFC), an area responsible for interpreting the emotional, or affective aspects of stimuli, particularly related to mental states. These participants performed well on classic second-order false belief tasks, but failed at irony and faux-pas detection. The researchers suggested that in order to understand irony, one must go beyond a cognitive appraisal of mental states to interpret the emotional aspects of the other's mind, acquiring insight into the “attitude” layer of understanding. This research supports the idea that emotional understanding is particularly important to understanding irony but, to our knowledge, this relationship has never been tested in irony appreciation for typically developing children.

Certainly, there is evidence that children's understanding of emotion and emotional expression develops over the same developmental timeframe as does irony appreciation (Pons et al., 2004). From 6 to 10 years emotion recognition becomes more sophisticated and also increasingly right-lateralized in typical development (Watling and Bourne, 2007). During this time frame, children also come to appreciate the possibility that emotions can be controlled or regulated (Gnepp and Hess, 1986). Irony is one such strategy. Further, there are individual differences in children's emotion recognition and emotional perspective taking skills (Cutting and Dunn, 1999), and some of this variability could be related to differences in irony appreciation.

### The process of irony appreciation

Much of the previous developmental research has evaluated the product of children's irony appreciation, rather than the process. Yet there are different theoretical frameworks for irony processing (for a review, see Gibbs and Colston, 2012) and it is important that we evaluate which of these is the better description of children's behavior.

On the one hand, a set of modular theories posit that a literal interpretation of the statement must be accessed first, before the perceiver can consider other, non-literal interpretations. This view stems from Grice's (1975) standard pragmatic approach. A prominent exemplar of this set of theories is the graded salience hypothesis (Giora, 1997), which suggests that since a literal interpretation is almost always the most salient meaning for a listener, it will be processed first. Only when it is judged that the literal interpretation does not fit within the context can the listener move on to a non-literal interpretation (see also Dews and Winner, 1999; Schwoebel et al., 2000). As such, understanding ironic meaning involves extra inferential processes, and should thus take longer, than understanding literal meaning.

On the other hand, the interactive approach suggests that ironic statements need not be processed in a serial fashion. Instead, ironic meanings can be accessed from the earliest moments of processing if ironic intent is congruent with the context (Gibbs, 1986). Parallel constraint satisfaction is a prominent interactive theory which suggests cues are processed rapidly and in parallel and an ironic interpretation is considered as soon as there is evidence that it might be appropriate (Katz, 2005; Pexman, 2008). As such, it should not necessarily take longer to process an ironic statement than a literal statement.

Typically these claims have been tested in reading time studies with adult participants. Some of these studies have found that participants take longer to read an ironic vs. a literal statement, suggesting that irony appreciation involves extra inferential processes as predicted by modular accounts (e.g., Dews and Winner, 1999; Filik and Moxey, 2010; Akimoto et al., 2012). In contrast, other reading studies have reported that, with supportive context, ironic statements can be processed just as fast and in some cases faster than their literal counterparts (e.g., Gibbs, 1986; Ivanko and Pexman, 2003), lending support to interactive theories.

One limitation of the reading time studies, however, is that while one can collect information about which type of statement takes longer to process, one does not learn much about why processing is slowed. That is, the method reveals differences in total processing time but does not provide insight about the cause of those processing differences. Recently, the visual world paradigm has emerged as a new method for studying processing, and one that provides more information about the nature of processing (Huettig et al., 2011). By this paradigm, it is assumed that “the mind is going where the eye is going” (Trueswell and Gleitman, 2004, p. 320). Thus, one can assess thinking processes through looking behavior.

For instance, Kowatch et al. (2013) devised a variant of the visual world paradigm to explore adults' irony processing. In what was described as “the shopping task,” a speaker delivered literal or ironic statements about what they wanted to buy (e.g., I just *love* apples) and participants were asked to select, from two objects placed on either side of a tabletop (e.g., an apple and an orange), the one the speaker wanted and to put it in a small shopping cart in the center of the tabletop. Participants' eye gaze to the objects was tracked by digital video, so the researchers could investigate not only how long it took participants to make their decisions about speaker intent, but also which meaning (object) they considered (looked to, reached for) during the decision making process. Results showed that ironic requests (I just *love* apples) were processed in the same time as literal negations (I just hate apples). Perhaps more importantly, after hearing an ironic remark participants tended to look first to the object corresponding to an ironic interpretation, and did so just as readily as they did to the object corresponding to a literal interpretation following a literal remark. Thus, there was no evidence of a bias to process the literal meaning first; ironic interpretations were considered in early moments of processing.

Since children do not usually have the reading skills required for reading time studies, the visual world paradigm provides a viable method of studying irony processing in children. In addition, the visual world paradigm minimizes verbal demands on child participants, as their response is manual (object selection) and they are not asked to justify their selection. Climie and Pexman (2008) devised the first such study. In their paradigm, 5- to 8-year-olds children watched a series of short puppet shows. In each show, children heard one puppet make a literal or ironic statement, and made a judgment about the speaker's intent by placing one of two toys in an answer box. Children were trained in advance to select one toy, the smiling yellow duck, if the speaker was being “nice,” and to select the other toy, a snarling shark, if the speaker was being “mean.” Climie and Pexman examined children's eye-gaze to the two response objects in order to gain insight about which meanings children considered during processing of literal and ironic statements. On hearing an ironic remark, children tended to look to the toy that corresponded with an ironic interpretation first, suggesting that processing of a literal interpretation is not obligatory, and that children seem to be considering an ironic interpretation very early in processing. These findings lend support to the constraint-satisfaction framework.

Although Climie and Pexman's (2008) findings were taken as evidence that children's irony processing is best described by an interactive theory, like the parallel constraint satisfaction framework, an important issue remains. In Climie and Pexman's procedure, children heard the speaker's statement and then were prompted to respond by the experimenter. Eye gaze data were gathered only after the experimenter's prompt. This left a gap of up to several seconds in processing time between the onset of the literal or ironic remark and the beginning of data collection. Yet it is possible that children began processing the ironic meaning at an earlier moment, as the ironic remark unfolded, and that if this early processing were assessed there would be evidence that the literal meaning was considered first. One goal of the present study was to devise a variant of the visual world paradigm that would allow assessment of the earliest moments of children's irony processing, in order to determine whether those moments are best characterized by a modular or interactive theory.

### The present study

In order to measure the earliest moments of children's processing of irony, we devised a modified version of Climie and Pexman's (2008) shark/duck task. In this new visual world task, the statements all took similar form (in the Climie and Pexman study the words in the target statements varied on each trial making it difficult to compare processing across statements) and children were trained to respond on each trial without a verbal cue from the experimenter. That is, in the context of short puppet shows, participants were presented with ironic statements (“that was *so* good”), literal criticisms (“that was so bad”), and literal compliments (“that was so good”). In this way processing for ironic remarks could be compared to that for a statement of opposite intent but the same wording (literal compliment) and opposite wording but similar intent (literal criticism). Children were trained in advance that, on each trial, if a speaker puppet made a positive evaluation of the target puppet's performance they were to pick up the duck and put it in the answer box, and if the speaker puppet made a negative evaluation they were to pick up the shark and put it in the answer box. Children's behavior was video recorded to allow analysis of their eye gaze and responses as each statement unfolded in real time. In addition, we separately assessed children's empathy and language skills in order that we could evaluate the relationship of these skills to irony appreciation and processing.

#### Predictions

If empathy skills are important to development of irony appreciation, then we predicted that children's empathy would be related to both the product and process of their irony appreciation. Consistent with previous research, children's judgments of speaker intent were expected to be more accurate for literal than for ironic statements (e.g., Harris and Pexman, 2003; Climie and Pexman, 2008). Children were also expected to be faster in responding to literal compliments given the advantage for processing positive literal statements found in previous research (Ivanko and Pexman, 2003; Climie and Pexman, 2008; Kowatch et al., 2013). For correctly interpreted trials, eye-gaze and reaction time data were analyzed to test predictions of modular and interactive theories of irony processing. According to modular accounts, children should consider the literal response object before the ironic object. Further, response times should be longer for ironic statements than literal statements of the same valence. In contrast, according to interactive accounts, children should consider the ironic response object early, without first considering the literal object, and their response times should be consistent across statement types of the same valence.

## Methods

### Participants

The goal of the present study was to examine irony appreciation in children on the cusp of understanding ironic language. Therefore, a group of thirty 6- and 7-year-olds were first tested, as we expected these children to be just beginning to make accurate assessments of speaker intent for ironic statements. This pilot testing revealed that in this particular task, this age group had near-zero accuracy for ironic statements. Consequently, a second child group of thirty-one 8- and 9-year-olds (17 female) was subsequently recruited and served as the main participant group. These children were from middle-class families in the Calgary area and were recruited through the Child and Infant Learning and Development (Ch.I.L.D.) database at the University of Calgary. Descriptive statistics for this group are presented in Table 1.

**Table 1:** **Participant characteristics**.

**Variable**	***M (SD)***
Age in months	110.48 (6.79)
CCC2 Structure	43.35 (6.72)
CCC2 Pragmatic	41.97 (7.33)
CCC2 Social	20.84 (4.65)
CCC2 Composite	85.68 (12.09)
EQ-C	36.97 (8.31)

CCC2 = Children's Communication Checklist-2 (Bishop, 1998); EQ-C = Empathy Quotient-Children (Auyeung et al., 2009).

All participants were required to have English as their first language in order to properly comprehend the procedure, and to have had no prior participation in irony-based studies at the University of Calgary in order to avoid priming effects or bias in the results. All participants were tested individually in an on-campus laboratory and received a small prize for participating.

### Materials

Participants were shown a series of twelve short puppet show scenarios (see examples in the Appendix). Each show consisted of two puppets and relevant props and depicted scenarios that should be familiar to children. There were a total of 24 puppets used in the puppet shows and two additional puppets were used in training. The puppets were dressed appropriately for the context of the show (e.g., wearing outdoor gear for a winter setting), and most stories involved the use of one or more props appropriate to the context (e.g., a snowboard, an easel for painting, a boat for water skiing).

Each of the puppet shows consisted of a short story involving two puppets, which ended in a statement uttered by one of the puppets. There were three different statement types: six ironic criticisms, three literal compliments, and three literal criticisms. In order to counterbalance so that each puppet show was in each statement condition at least once, four versions of the procedure were created. In this way, across versions (and thus across participants), each puppet show was presented in each literal condition once and in the ironic condition twice. The order of shows for each version was randomized using an online random list generator. These lists were then manually manipulated so there were no more than two instances of the same statement type in a row (e.g., no more than two consecutive ironic criticisms). Additionally, the order was manipulated so that in each version, the first puppet show was a literal statement in order to provide an intonation baseline; this allowed the participant to better understand the speaker's typical (i.e., sincere) tone of voice so that they could detect any later intonation differences that might signal an ironic tone of voice. Two versions began with a literal compliment and two versions began with a literal criticism.

Participants were asked to make a judgment about the target statement that came at the end of each puppet show. To do this, participants were instructed to “show” the researcher their answer by placing one of two toys—a “nice” duck and a “mean” shark—in an answer box. Both were soft, plush toys, approximately 3–4″ in length.

**Figure 1:**
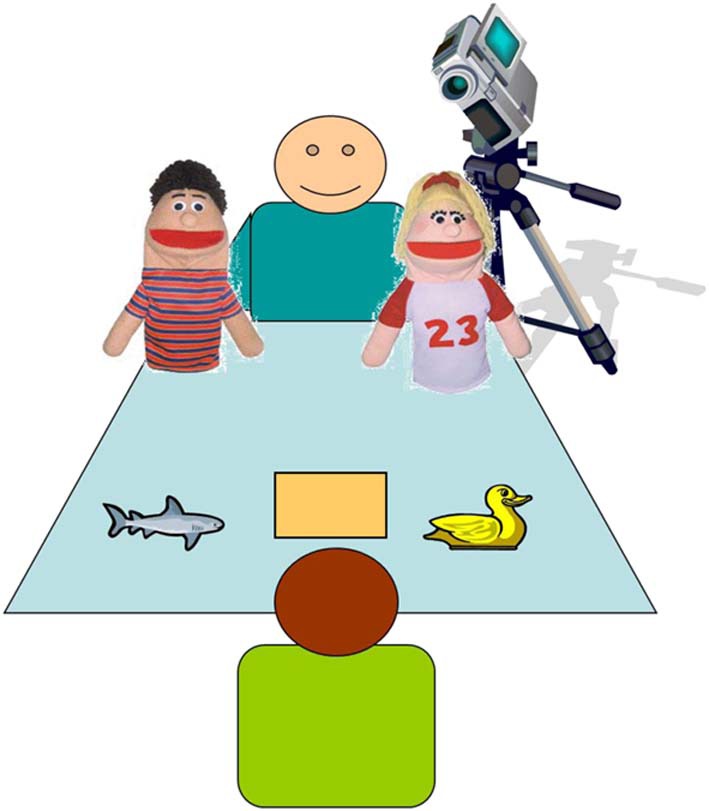
**Testing set up**.

The experimenter and participant sat across from each other at a table, which was set up as follows: the shark and duck toys were placed at the edge of the table in front of the participant, one on the right side, and one on the left side. For the duration of the study, the location of the shark and duck stayed the same, but their location was counterbalanced across participants. The answer box in which the toy would be placed was in the middle of the table, directly in front of the participant. For a visual depiction of this set-up, see Figure 1.

Audio narration for the puppet shows was pre-recorded and then played during the puppet shows so the narration was not coming from the experimenter. The recorded speaker was of the opposite gender to the experimenter to minimize likeness with the experimenter. This was done to reduce the chance that the participant would look to the experimenter for clarification, as this would interfere with eye-gaze data.

To achieve consistency across trials the same statement was recorded for each puppet show: “That was so good” for literal compliments and ironic criticisms, and “That was so bad” for literal criticisms. The critical statements for each of the three conditions were similar in length (literal compliment: 1400 ms; literal criticism: 1567 ms; ironic criticism: 1433 ms). The critical word (“good” or “bad”) was uttered at a similar time point in each statement (literal compliment: 933 ms; literal criticism: 1033 ms; ironic criticism: 1067 ms). A separate group of five adults who were blind to the study purpose were asked to rate multiple samples of these statements on how literal or sarcastic they were, based on prosody alone, without the context of the story. The highest rated exemplars of each statement type were then spliced into each corresponding puppet show recording. Children's eye gaze and reaching behaviors during the puppet shows were recorded using a digital video camera sitting on a tripod positioned over the experimenter's shoulder, keeping the child and the response objects in frame.

Parents were asked to fill out two questionnaires. The first questionnaire was the empathy measure, the Empathy Quotient for Children (EQ-C), which has been shown to have good reliability and validity (Chapman et al., 2006; Auyeung et al., 2009). The second questionnaire was the Children's Communication Checklist—2 (CCC2), a standard communication checklist that has also been shown to have good reliability and validity (Bishop, 1998). We selected the CCC2 to estimate children's language and communication skills because it includes three aspects of communicative competence: language structure (subscales A, B, C, and D), pragmatic (subscales E, F, G, and H), and social (subscales I, J) dimensions. Children's mean EQ-C and CCC2 (scaled) scores are presented in Table 1.

### Procedure

The procedure involved several training trials, which highlighted the use of the “nice” duck and the “mean” shark. Children were trained to choose one of these objects, depending on whether the participant interpreted the puppets' statements as nice or mean. It was emphasized that children should listen very carefully to the puppet shows, and then make their decision without the researcher giving a prompt. They were instructed to choose their answer immediately following the conclusion of the puppet show, placing either the shark or the duck in the answer box. The participant practiced this procedure with the two practice puppets for a maximum of four training puppet show scenarios (2 literal compliments, 2 literal criticisms, all different statements than those used in the test trials) before beginning the experimental trials.

### Coding

Participants' selections of the response objects were coded as either correct or incorrect. Correct responses consisted of choosing the shark for literal criticisms and ironic criticisms and choosing the duck for literal compliments. If a verbal response was given (1.08% of literal compliments, 2.15% of literal criticisms, and 1.61% of ironic criticisms), we coded only accuracy and not latency. Using the video recording, eye gaze, and behavioral response (arm-reach) latencies were coded frame-by-frame by the experimenter. Arm reach behavior was coded in three phases in order to examine early, middle, and late processing (Climie and Pexman, 2008; Kowatch et al., 2013): (1) Early Phase: from the beginning of the spoken critical statement (e.g., *that* in “that was so good.”) to the initiation of hand/arm movement by the participant; (2) Middle Phase: from the initiation of the hand/arm movement to when the participant made contact with their choice of response object; and (3) Late Phase: from the touch of the response object to when the object was placed in the answer box. Eye gaze was measured for the first object that was fixated following the critical statement, and for the proportion of looking time to the correct response object.

A second coder, who was blind to the experimental purpose or hypotheses, coded 25% of the video recordings. Agreement between coders was acceptable: Early phase timing: intraclass correlation coefficient (ICC) = 0.99, Middle phase timing: ICC = 1.00, Late phase timing = 1.00, Looks to shark: ICC = 0.98, Looks to duck: ICC = 0.98, Time looking to shark: ICC = 0.98, Time looking to duck: ICC = 0.99.

## Results

### Accuracy

**Figure 2:**
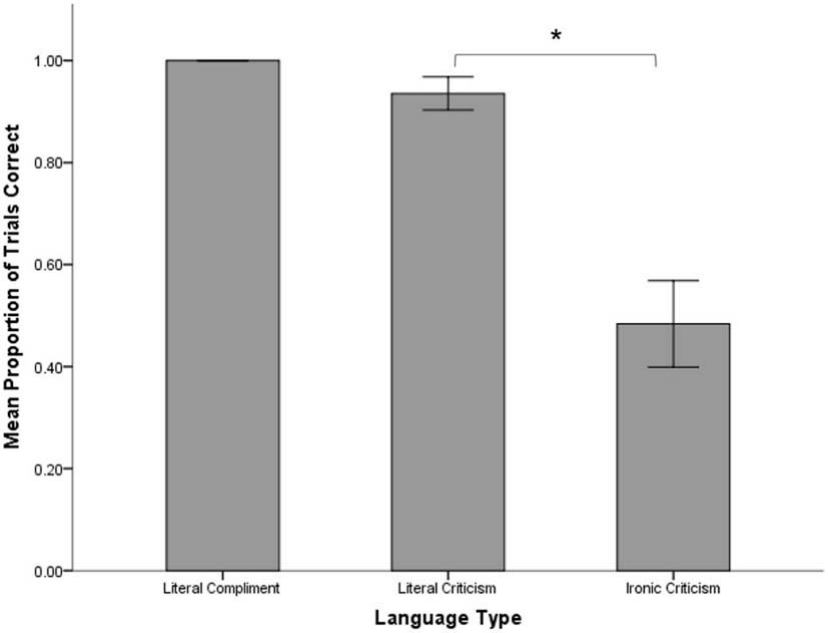
**Mean proportion of trials with correct speaker intent actions across all three language types.** Bars represent standard error. Asterisk represents a significant difference in mean accuracy (*p* < 0.001).

The proportions of trials on which participants responded correctly in their assessment of speaker intent were examined for each of the three statement types (Figure 2). Participants were at ceiling in accuracy for literal compliments (*M* = 1.00, *SD* = 0.00), so this condition was not included in further accuracy analyses. A paired samples *t*-test was conducted on the accuracy scores for the remaining two statement types, ironic criticisms and literal criticisms, and this showed that children were significantly less accurate in their speaker intent actions for ironic criticisms (*M* = 0.48, *SD* = 0.47) than for literal criticisms (*M* = 0.94, *SD* = 0.18), *t*_(30)_ = 4.55, *p* < 0.001.

### Response latencies

Arm-reach data was split into three phases in order to gain more detailed information about the time-course of decision-making (Climie and Pexman, 2008; Kowatch et al., 2013). Any trials on which the response latency was more than 2.5 standard deviations above the mean for each participant were removed as outliers (<5% of trials). A summary of response latencies can be found in Table 2.

**Table 2:** **Mean response latencies (ms) for each phase of arm reach during speaker intent actions**.

	**Early phase**	**Middle phase**	**Late phase**	**Total**
**CORRECT RESPONSES**				
Literal compliment	2022.88 (464.33)	553.59 (146.04)	754.90 (148.53)	3226.34 (858.03)
Literal criticism	2771.90 (737.72)	607.19 (165.84)	975.49 (658.10)	3879.39 (1050.40)
Ironic criticism	2788.04 (1168.27)	516.56 (126.70)	809.08 (212.46)	4113.68 (1389.53)
**INCORRECT RESPONSES**				
Ironic criticism	2905.82 (1894.20)	537.87 (129.48)	932.28 (378.45)	4375.97 (2175.79)

Values in parentheses indicate standard deviation.

Within-subjects One-Way ANOVAs were conducted to examine the response latencies for correct actions across the three statement types, for each response phase. A significant effect of statement type was found in the early phase, *F*_(2, 32)_ = 5.97, *p* < 0.05, *MSE* = 544473.12. Follow up comparisons (adjusted for multiple comparisons with the Bonferroni correction) revealed children were significantly slower to initiate a reach to the correct object in the literal criticism condition (*M* = 2771.90 ms, *SD* = 737.72) than in the literal compliment condition (*M* = 2022.88 ms, *SD* = 464.33), *t*_(30)_ = 4.54, *p* < 0.017. Additionally, children were significantly slower responding to ironic criticisms (*M* = 2788.04 ms, *SD* = 1168.27) than to literal compliments, *t*_(16)_ = 2.96, *p* < 0.017. There was no significant difference found in response latencies for ironic criticism and literal criticism conditions, *t*_(16)_ = 0.05, *p* = 0.96.

There were no significant differences in response latency as a function of statement type in either the middle phase, *F*_(2, 32)_ = 2.96, *p* = 0.07, or the late phase of responses, *F*_(2, 32)_ = 1.37, *p* = 0.27. Therefore, the only difference in processing speed reflected in response latencies emerged in the earliest phase of processing.

### Eye gaze

**Figure 3:**
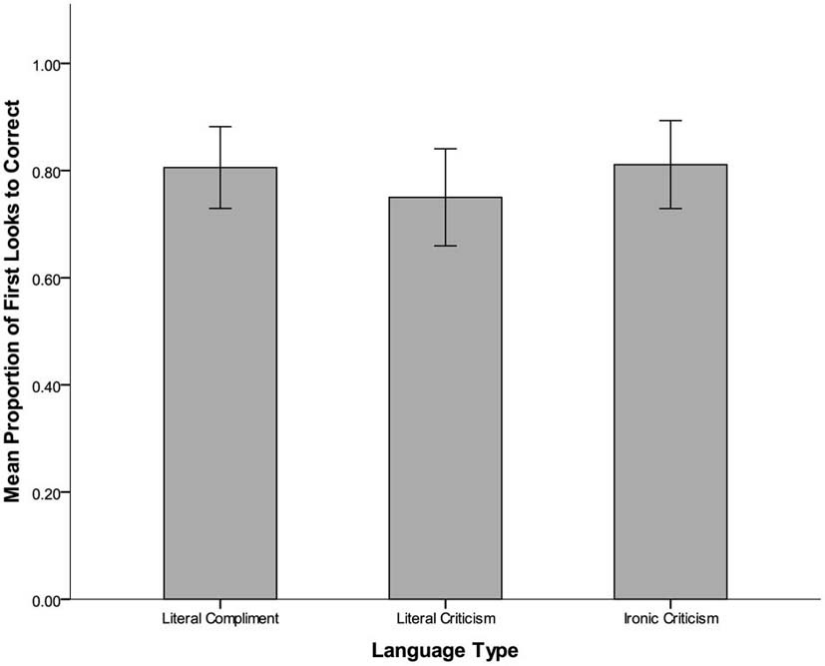
**Mean proportion of first looks to the correct object on correct trials.** Bars represent standard error.

For correct trials, proportions of first looks to the correct response object (vs. the incorrect response object) were examined in order to gain insight into which response alternative children considered in the early moments of processing (Figure 3). A within-subjects One-Way ANOVA revealed no significant effect of statement type, with equivalent proportions of first looks for literal compliments (*M* = 0.90, *SD* = 0.20), literal criticisms (*M* = 0.85, *SD* = 0.29), and ironic criticisms (*M* = 0.84, *SD* = 0.27), *F*_(2, 22)_ = 0.19, *p* = 0.83. These data show that children tended to look first to the correct object as they initiated their responses to ironic statements, and did so just as often when responding to literal statements.

Although children did not show any tendency to look to the literal response object first as they judged speaker intent for ironic statements, we also examined their looking behavior across the whole response to test whether there was evidence for a literal bias for ironic statements in later looking behavior. The proportion of time spent looking at the correct object across the entire response was examined with a within-subjects One-Way ANOVA. Results showed no significant effect of statement type on proportion of looking time to the correct object, *F*_(2, 22)_ = 0.35, *p* = 0.71. The proportion of looks to the correct object was equivalent across literal compliment (*M* = 0.86, *SD* = 0.18), literal criticism (*M* = 0.90, *SD* = 0.13) and ironic criticism (*M* = 0.85, *SD* = 0.18) statement types.

For incorrectly interpreted ironic criticisms, children showed little tendency to look first to the shark (the correct response object, *M* = 0.10, *SD* = 0.24) and spent only a small proportion of time looking at the shark across the whole response (*M* = 0.07, *SD* = 0.14). Thus, at least in their looking behavior, there was little evidence that children who responded incorrectly had implicit understanding of ironic intent.

### Irony and empathy

In order to examine relationships between irony appreciation and empathy, we first conducted correlation analyses (Table 3). Results showed, not surprisingly, that children's scores on the CCC2 social subscale were positively related to their scores on the EQ-C measure. Further, results showed that none of the language subscales, composite scores, or children's age (all factors that we anticipated we might need to control in analyses of the relationship between empathy and irony) were correlated with either empathy or irony appreciation measures. As such, the correlations could be interpreted without additional analyses controlling for age or CCC2 scores. Notably, the results included several significant correlations between aspects of children's irony appreciation and empathy. Higher empathy scores were associated with a tendency to look first to the correct object following ironic statements, and with a higher proportion of looks to the correct object when appraising ironic intent. In addition, higher empathy scores were associated with children's accuracy on the speaker intent response action. The speaker intent action accuracy data were not normally distributed, however, as children tended to be either quite accurate (above chance performance on this forced-choice response, *n* = 15) or quite inaccurate (below chance performance on this forced-choice response, *n* = 16). As such, we opted to treat this aspect of children's irony understanding as a categorical variable and as a dependent measure in a logistic regression. Children's empathy was used as the only predictor and results showed a significant relationship between children's empathy and their speaker intent action accuracy, *B* = 0.14, *SE* = 0.07, *p* = 0.041, with stronger empathy associated with more accurate speaker intent actions.

**Table 3:** **Correlations among study variables**.

**Variable**	**1**	**2**	**3**	**4**	**5**	**6**	**7**	**8**	**9**	**10**	**11**	**12**	**13**	**14**	**15**	**16**	**17**	**18**
1. Age	−																	
2. CCC2 structure	0.27	−																
3. CCC2 pragmatic	0.15	0.64^***^	−															
4. CCC2 social	0.08	0.32	0.62^***^	−														
5. CCC2 composite	0.22	0.89^***^	0.90^***^	0.47^**^	−													
6. EQ-C	0.18	0.19	0.32	0.54^**^	0.29	−												
7. Speaker intent accuracy	0.15	−0.02	0.23	0.14	0.16	0.43^*^	−											
8. First look correct	−0.06	0.35	0.06	−0.08	0.27	0.76^***^	0.09	−										
9. Proportion looks correct	−0.18	0.36	0.07	−0.16	0.28	0.69^**^	0.23	0.93^***^	−									
10. First look incorrect	−0.04	−0.30	−0.34	−0.17	−0.34	0.12	0.25	0.28	0.34	−								
11. Proportion looks incorrect	−0.03	−0.42	−0.43	−0.35	−0.41	0.07	0.23	0.31	0.38	0.96^***^	−							
12. Early phase RT—correct	0.03	−0.19	−0.14	−0.02	−0.18	−0.02	−0.37	−0.04	−0.07	−0.02	−0.01	−						
13. Middle phase RT—correct	0.07	−0.47	−0.15	−0.16	−0.33	−0.10	0.32	−0.31	−0.36	0.02	0.05	0.47	−					
14. Late phase RT—correct	0.06	−0.21	−0.06	−0.11	−0.15	−0.20	−0.17	−0.20	−0.26	0.06	0.05	0.65^**^	0.79^***^	−				
15. Total RT—correct	0.05	−0.24	−0.14	−0.05	−0.20	−0.05	−0.31	−0.09	−0.13	0.00	0.01	0.98^***^	0.61^**^	0.77^***^	−			
16. Early phase RT—incorrect	0.01	0.04	0.24	0.24	0.13	0.49^*^	0.32	0.30	0.39	0.04	−0.01	0.05	−0.24	−0.31	−0.02	−		
17. Middle phase RT—incorrect	−0.25	−0.21	0.05	−0.07	−0.08	0.04	0.01	0.27	0.31	−0.18	−0.12	0.51	0.02	0.06	0.44	0.46^*^	−	
18. Late phase RT—incorrect	−0.26	−0.38	0.12	0.33	−0.16	0.34	0.26	0.34	0.37	0.09	0.10	0.59^*^	0.05	−0.01	0.51	0.49^*^	0.65^**^	−
19. Total RT—incorrect	−0.05	−0.05	−0.05	0.26	0.08	0.49^*^	0.33	0.32	0.16	0.04	0.00	0.13	−0.21	−0.27	0.05	0.98^***^	0.58^**^	0.64^**^

* p < 0.05,

** p < 0.01,

*** p < 0.001.

Additional insight is provided by the correlations for measures of children' incorrectly interpreted ironic remarks. That is, when children incorrectly interpreted speaker intent for ironic criticisms, their response latencies (in particular, their early phase response latencies) were correlated with their empathy scores, such that children with higher empathy scores responded more slowly on incorrect trials. This may suggest that children with stronger empathy had greater implicit understanding of ironic intent, and that understanding was strong enough to slow their response but not strong enough to produce a correct speaker intent action. In sum, the correlational results suggest that empathy is related to both comprehension and processing of ironic remarks, even when those remarks are interpreted incorrectly.

## Discussion

The purpose of the current study was to investigate the product and process of children's irony appreciation, and the role of empathy in those aspects of communicative development. Previous research examined children's processing of irony, but used measures that left a gap of several seconds in which important cognition could already have taken place (Climie and Pexman, 2008). The current study addressed this gap with a modified procedure that allowed examination of both comprehension and processing of ironic vs. literal statements in the earliest moments, as the utterance was unfolding. Results address whether children are utilizing a modular or interactive approach to processing, and also draw links with other aspects of children's emotional development.

In their appraisals of speaker intent, results showed that children were at, or near, perfect performance on both literal compliments and criticisms. This suggested that literal statements are much easier for children to understand than ironic statements. Children's speaker intent accuracy for ironic statements was at 48%, significantly lower than accuracy in the literal criticism condition. While this level of performance could be interpreted as equivalent to chance (since there were two response options), many irony researchers have argued that because literal language is vastly more common than irony in everyday speech, the appropriate comparison for irony is not to chance; rather, one can assume that the literal interpretation is more common and any deviation from the literal interpretation is meaningful. Further, examination of children's speaker intent accuracy showed that they tended to be either very accurate or not at all accurate. In this light, the children's speaker intent accuracy for ironic criticisms suggests that some children in this age group are able to appreciate ironic language. This suggests, further, that this age group, in which children seem to be on the cusp of irony appreciation (at least in present task conditions), is suitable for our investigation of irony processing. That is, if any children were inclined to show a literal bias in processing one would think it would be children who are just beginning to understand ironic language.

As expected, children's response latencies showed a positive advantage, in that they were faster to respond to positive statements (literal compliments) than negative statements (literal criticisms and ironic criticisms; Ivanko and Pexman, 2003; Climie and Pexman, 2008; Kowatch et al., 2013). The present results show, further, that this positive advantage emerged in the early phase of responding. When processing of ironic remarks was compared to processing of literal remarks of the same valence (literal criticisms), however, the processing times were equivalent. Modular theories suggest that listeners follow a stage-like process of interpreting and then rejecting a literal interpretation before moving on to an ironic interpretation; as such, processing should take longer for ironic criticisms than for literal criticisms (Dews and Winner, 1999). Children in the present study were not slower responding to ironic vs. literal criticisms, suggesting that processing irony does not necessarily involve extra inferences, consistent with interactive theories (Pexman, 2008).

Eye-gaze behavior provided further insight. Children's first looks were examined to investigate the meanings that children were initially considering as they made their speaker intent judgments. Results showed that for ironic statements that were correctly appraised, children looked to the correct, ironic response object the majority of the time, and did so just as often as they looked to the correct, literal response object following literal statements. As such, first look data suggest children do not need to consider a literal interpretation of an ironic utterance and then reject the literal interpretation in favor of an ironic interpretation, as modular theories would posit (e.g., Giora, 1997). Instead, children are able to consider an ironic interpretation from the earliest moments (Katz, 2005; Pexman, 2008). Beyond the first look, we also examined the proportion of time children spent looking at the correct object. Across children's responses we found no significant differences between the literal conditions and the ironic condition, suggesting that children do not give extra consideration to the literal meaning when processing ironic statements. As such, children's eye gaze behavior provides support to interactive theories of irony processing.

In order to explore the relationship of irony comprehension and processing to emotional perspective-taking skills, correlational analyses were conducted. Results showed that children's speaker intent action accuracy, first looks to the correct object, and proportion of looks to the correct object following correctly interpreted ironic statements were each significantly correlated with children's scores on the EQ-C, a measure of empathy. Further, there was evidence that children's empathy scores were related to their implicit awareness of ironic intent for incorrectly interpreted ironic remarks. As such, the present results confirm claims made by Shamay-Tsoory et al. (2005) about the importance of empathy or emotional perspective taking to irony understanding, but here we tested them directly. Results showed that children who were judged by their parents to have relatively strong empathy skills were, in our shark/duck task, able to judge speaker intent for irony more accurately, and tended to look first and for a greater proportion of their response time to the correct response object. These findings provide important new clues about how irony detection and processing fit into the larger context of children's emotional development. One interpretation of our findings is that the reason irony appreciation is slow to develop (extending into adolescence) is because it requires advanced skills in assessing the affective consequences of others' language and an ability to detect attitudes even when they are conveyed indirectly.

To our knowledge, the present study is the first to consider associations between children's irony appreciation and empathy skills, and also the first to measure the earliest moments of children's processing of ironic speech. Certainly, there are limitations to the present work. In order to study processing we created a communicative environment that was impoverished as it lacked many of the cues that would be available in everyday language, such as facial expression and knowledge of the speaker. In addition, our study was limited to ironic criticisms, and did not consider children's appreciation of the less common but related form known as ironic compliment (i.e., a literally negative statement intended as praise, see e.g., Pexman and Glenwright, 2007). Further, we assessed children's empathy skills indirectly and with a unidimensional measure, by asking parents to rate children's empathy skills on a standard scale. The fact that we observed relationships between parents' ratings and children's interpretive behavior even under these circumstances suggests that a strong association may be present, one that should be investigated in a more fine-grained way in future research. In particular, it will be important to identify which aspects of empathy (e.g., cognitive vs. affective) are most strongly associated with irony appreciation.

## Conclusions

In the present study we modified the visual world paradigm to tap the earliest moments of children's processing during the unfolding of a spoken ironic statement. Results from response latency and eye gaze measures support an interactive framework, suggesting that, like adults, children consider an ironic interpretation in the earliest phases of processing. Empathy was strongly associated with several aspects of irony comprehension and processing, suggesting that emotional reasoning abilities are important to development of irony comprehension. By establishing this link between emotional development and irony comprehension in typical development we corroborate findings from studies of populations who struggle with both of these domains, such as children with Autism Spectrum Disorder (Pexman et al., 2011), and brain-damaged patients (Shamay-Tsoory et al., 2005). Appreciation of speaker intent for ironic language is a remarkable developmental achievement, and one that highlights the increasing sophistication of children's emotion recognition skills in middle childhood.

### Conflict of interest statement

The authors declare that the research was conducted in the absence of any commercial or financial relationships that could be construed as a potential conflict of interest.

## Appendix

### Example puppet show scenarios

#### The soccer scenario

Shannon and John play on a soccer team together. It is the last few minutes of a game. Shannon kicks the ball, (scoring a goal in the last minute of the game/missing the net entirely). John says:

Literal Compliment: That was so good.
Literal Criticism: That was so bad.
Ironic Criticism: That was so good.

#### The dinner scenario

Gary and Lucy are eating dinner one night. For dessert, Lucy brings out a cake that she had baked earlier in the day. The cake tastes (delicious/terrible). Gary says:

Literal Compliment: That was so good.
Literal Criticism: That was so bad.
Ironic Criticism: That was so good.
